# Next Generation Winemakers: Genetic Engineering in *Saccharomyces cerevisiae* for Trendy Challenges

**DOI:** 10.3390/bioengineering7040128

**Published:** 2020-10-14

**Authors:** Patricia Molina-Espeja

**Affiliations:** Department of Biocatalysis, Institute of Catalysis, CSIC, Cantoblanco, 28049 Madrid, Spain; patricia.molina@icp.csic.es

**Keywords:** genetic engineering, *Saccharomyces cerevisiae*, wine

## Abstract

The most famous yeast of all, *Saccharomyces cerevisiae*, has been used by humankind for at least 8000 years, to produce bread, beer and wine, even without knowing about its existence. Only in the last century we have been fully aware of the amazing power of this yeast not only for ancient uses but also for biotechnology purposes. In the last decades, wine culture has become and more demanding all over the world. By applying as powerful a biotechnological tool as genetic engineering in *S. cerevisiae*, new horizons appear to develop fresh, improved, or modified wine characteristics, properties, flavors, fragrances or production processes, to fulfill an increasingly sophisticated market that moves around 31.4 billion € per year.

## 1. Introduction

**Figure 1:**
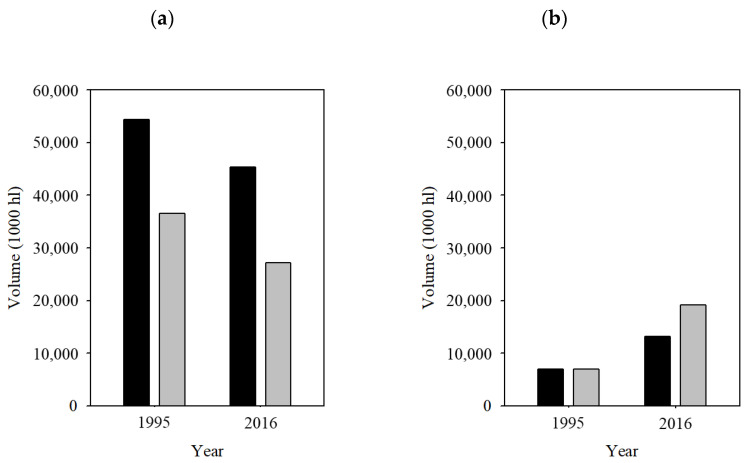
Differences from 1995 to 2016 in production and consumption of wine of two countries: (**a**) France is traditionally one of the largest wine producers and consumers of the world, and (**b**) China is an emergent country in this industry. Black bars: wine production; grey bars: wine consumption. Source: International Organization of Vine and Wine (OIV).

Open the bottle, fill the glass (the appropriate glass, the appropriate amount), smoothly remove it to oxygenate, observe the tears, close your eyes, smell it, and then, and only then, taste it. The ritual of wine. Looks simple, but behind these sensations there is a myriad of components that are continuously being sought for perfection. In the center of it all. *Saccharomyces cerevisiae*. This yeast is mainly responsible of the process of winemaking. As far as we know, humankind has been using *S. cerevisiae* for around 8000 years [1,2] although obliviously, for the production of bread, beer and wine. All along this time there has been a domestication of the yeast [3,4], although, again, without even being aware of it. Winemakers have been selecting for *S. cerevisiae* strains to improve flavor, fragrances, better performance, etc. It was 130 years ago that Emil Hansen at the Carlsberg Laboratory isolated for the first time the yeast responsible for beer fermentation, and his lead was followed by wine production [5,6]. Since then, and by the hand of biotechnological developments, such a “domestication” has experienced an intended boost. Now we know the chemical reactions that transform the sugars in the must into ethanol and CO_2_ by alcoholic fermentation, we know metabolic reactions that *S. cerevisiae* performs in the meanwhile, and how those affect the final product. We also know the genome of our favorite yeast: it was the first eukaryotic genome in to be fully sequenced in 1996 (strain S288C) [7], and keeps being updated at the *Saccharomyces* Genome Database (www.yeastgenome.org). Since then, sequencing efforts have been shedding light on the history of our yeast. For instance the sequencing of 38 *S. cerevisiae* strains from different sources (laboratory, pathogenic, baking, wine, food spoilage, natural fermentation, sake, probiotic and plant isolates) were analyzed, including in the study the strain S288C as reference [8]; 235,127 single-nucleotide polymorphisms (SNPs) and 14,051 insertions/deletions (indels) were disclosed, while five lineages were identified. More recently, the analysis of around 1000 natural *S. cerevisiae* isolates found 1,625,809 SNPs and 125,701 indels, most of them grouped in 26 clades [9]. All this knowledge is put to the service of an industry that produced 292 million hL and moved 31.4 billion € in 2018 worldwide, according to the International Organization of Vine and Wine (OIV, www.oiv.int). Although there is an economic stability in this market, there are variations in the interest put on wine by different countries. For example, France is lowering the production and consumption of wine, while China is an emergent country in this field (Figure 1).

**Figure 2:**
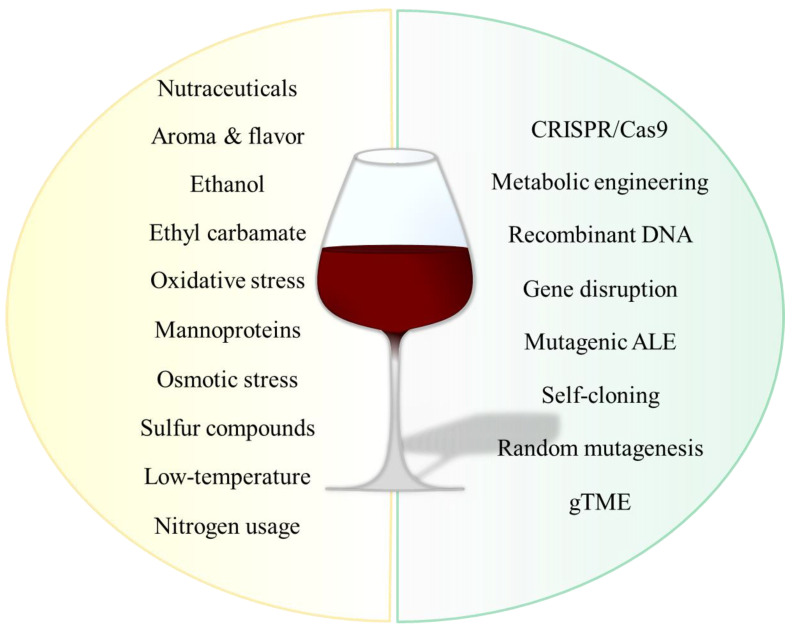
Genetic engineering of winemaking using *S. cerevisiae* as a tool and producer organism. On the left, the main traits targeted; on the right, the main genetic engineering techniques employed for this end.

Biotechnology and *S. cerevisiae* walk hand in hand-in-hand numerous aspects of industry: from the use of the yeast as a factory to produce for, instance, insulin or bioethanol [10,11,12,13,14]; Its use to improve enzymes by directed evolution or rational design [15,16,17]; its relevance as a model organism in genetics, molecular biology and physiology [18,19]; or in synthetic biology, for instance, by the ambitious project Yeast 2.0 (www.syntheticyeast.org) [20], to name a few. In particular, the genome of the commercial strain EC1118 (Lalvin EC1118, also known as Premier Cuvee or Prise de Mousse) has been fully sequenced and is an essential player in the issue at hand, since it is commonly employed as a model for understanding the process and as a winemaking fermentation starter [21]. This review focuses on recent and relevant works in genetic engineering employing *S. cerevisiae* for the production of wine based on current traits to be improved, modified or added (Figure 2).

## 2. Nutraceuticals

In past decades, several wine components have been targeted as beneficial for human health. Some of them are resveratrol, tyrosol, hydroxytyrosol, melatonin, serotonin, caffeic acid, tryptophol, glutathione or trehalose [22]. Here, focus is directed mainly to resveratrol and hydroxytyrosol, which show promising results.

### 2.1. Resveratrol

When stress appears due to fungal infection, UV radiation or wounds, molecules such as resveratrol (3,5,4′-trihydroxystilbene) are produced by grape berries, mainly in their skin [23]. To obtain red wine, the fermentation proceeds in the presence of skin, acquiring in the process values of up to 14.3 mg/L resveratrol [24]. In the case of white wine, 3- to 10-fold lower concentration of resveratrol is produced, since the fermentation occurs usually in the absence of the grape berry skin [25]. Beneficial effects of this polyphenol on health, even in micromolar amounts [26], include cardio-vascular protection, antioxidant and anti-inflammatory activity, anticancer properties, inhibition of platelet aggregation, lowering of blood glucose, protection against neurodegenerative diseases, obesity and osteoporosis, and also showed positive effects on fertility, lungs, visual, hepatic and renal systems [27]. It has been calculated that the intake of wine to reach therapeutic doses of resveratrol is, in the best case, 505 L per day [25]. To avoid such a hangover, approaches to increase the amount of this nutraceutical in wine have been tackled. For instance, resveratrol can be synthetized from malonyl-CoA and *p*-coumaroyl-CoA by the resveratrol synthase. Malonyl-CoA is already present in *S. cerevisiae* while *p*-coumaroyl-CoA can be formed from coumaric acid, also present in our yeast. However, this latter step needs the coenzyme-A ligase which is absent in *S. cerevisiae*. Thus, by inserting the coenzyme-A ligase gene from a hybrid poplar (4CL216) and the resveratrol synthase (RS) gene from grapewine (vst1) into yeast expression cassettes, and their expression in a laboratory strain of *S. cerevisiae*, resveratrol was successfully produced [28]. In another work, these genes (for 4CL, from *Arabidopsis thaliana* and RS from *Vitis vinifera*) were also included in the yeast strain EC1118, and in this case the culture media was supplemented with 1 mM *p*-coumaric acid. The outcome was a higher production of resveratrol when antibiotics (200 mg/L G418, 200 mg/L hygromycin) were added to the culture medium: 8.249 mg/L vs. 3.317 mg/L when absent [29].

In this line, the genes codifying phenylalanine ammonia lyase, cinnamic acid 4-hydroxylase, 4-coumarate: coenzyme-A ligase and stilbene synthase from different organisms were heterologously expressed in *S. cerevisiae* in order to fulfill and improve the metabolic pathway to resveratrol [30]. Since malonyl-CoA can be limiting, the GAL1 promoter (P_GAL1_) was introduced instead of the native promoter of the acetyl-CoA carboxylase, resulting in an overexpression of the enzyme. All this, plus the supplementation with tyrosine, led to the production of 5.8 mg/L of resveratrol.

As an aside before continuing with our theme, it can be noted that throughout all the text, the format of genes and proteins can seem inconsistent. This is due to the different origins of the elements: the organisms of which genes and proteins are obtained belong to different groups that have different rules. For instance, regarding the format for yeast, the convention is to use upper case letters for genes and lower case letters for proteins. For bacteria, genes are written in lower case and proteins with initial upper case. Here, italics have been avoided for genes and proteins.

### 2.2. Hydroxytyrosol

The main source in diet of this phenylethylalcohol is extra virgin olive oil, followed by wine [31], where its concentration ranges from 0.28 to 9.6 mg/L [32]. Following the Ehrlich pathway, tyrosine is transformed into tyrosol during alcoholic fermentation by yeast [33], which is the hydroxytyrosol precursor. In this section the focus is directed to hydroxytyrosol since it seems to have stronger antioxidant power than tyrosol [34]. The higher amount of hydroxytyrosol reported in wine is 25 mg/L [35], while for tyrosol is 31.62 mg/L [36]. Possitive effects on health of hydroxytyrosol have been listed as antioxidant, antiatherogenic and cardioprotective, osteoprotective, anticancer, antimicrobial and antiviral, neuroprotective, antidiabetic, lipid-regulating and antiobesity, as well as having beneficial results in inflammatory diseases, immune system, protective effect on UV-B in skin, and in renal hypoxia [34,37]. In sight of its potential, production of hydroxytyrosol has been promoted by introducing the hydroxylase HpaBC complex from *Escherichia coli* in *S. cerevisiae* [32]. The best results were 4.6 mg/L with supplementation of 1 mM of tyrosol while the control roughly reached 40 µg/L. A kind of “reverse” experiment consisted in expressing the genes codifying the ketoacid decarboxylase ARO10 and the alcohol dehydrogenase ADH6 from *S. cerevisiae* and overexpressing the native HpaBC complex in *E. coli* which led, together with media optimization, to a production of 647 mg/L of hydroxytyrosol from simple carbon sources [38]. Other studies have been centered in improving toluene monooxygenases (TMOs) by directed evolution and rational design to produce hydroxytyrosol via double hydroxylation of 2-phenylethanol [39]. Although this work is an example that uses *E. coli* as heterologous host, such approach could be transferred to *S. cerevisiae* wine strains, gaining in one step the production of the beverage and the nutraceutical. Other hydroxylative enzymes could follow this lead and be evolved to produce hydroxytyrosol from tyrosol such as cytochrome P450 monooxygenases or unspecific peroxygenases [40,41,42].

This interesting line of investigation on nutraceuticals can also take advantage of “waste” material from the winemaking process such as pomace or grape seeds, which accumulate a great amount of potential compounds with benefits for wellness [43]. Besides, tryptophol and tyrosol (together with 2-phenylethanol, amongst others) have been seen to be involved in quorum sensing in the yeast, which can open a useful line of investigation for improving wine fermentation, for instance, applying genetic engineering on the genes ARO8, ARO9, and ARO10, which are implied in the synthesis of these molecules [44,45].

## 3. Aroma and Flavor

In the course of yeasts performance towards wine, its action can influence the aroma composition (one of the most important characteristics of wines) [46] by de novo generation of primary or secondary metabolites, or by modifications such as the release of moieties of molecules whose “kidnapping” was hiding its scent [47]. We know how smell can influence flavor, and the best wine-tasters are capable of distinguish varieties and qualities of wine almost only with their nose. To innovate in this wine attribute, different strategies have been faced, such as has been recently reviewed [47]. Here, some interesting examples are depicted.

Aromatic thiols such as 3-mercaptohexan-1-ol (3MH) show grapefruit, passion fruit, and boxwood flavors, and can be produced along wine fermentation. *E. coli* tnaA gene codifies a tryptophanase with strong cysteine-*β*-lyase activity, which can produce 3MH from its non-volatile cysteinylated precursors Cys-3MH. This gene was cloned and overexpressed in a wine yeast strain under the control of the yeast constitutive promoter PGK1 (phosphoglycerate kinase I, P_PGK1_). This modified strain was able to release up to 25-fold more 3MH under fermentation conditions than the control host strain [48]. In a similar approach, it was proved that the native Srt3p enzyme showed activity on Cys-3MH in fermentative wine processes. The commercial yeast strain VIN 13 was engineered to integrate an additional copy of the native STR3 *S. cerevisiae* gene under the control of the P_PGK1_, in order to increase its expression [49]. The modified yeast strain released as a result 278 ng/L of 3MH in fermentation, an amount 27% higher than the control, while pH, glycerol, ethanol and organic acid production were preserved.

Another sensory molecule that has attracted interest is raspberry ketone [4-(4-hydroxyphenyl)butan-2-one]. This phenylpropanoid can be found in fruits, berries and vegetables, such as grapes, in quantities of 1–4 mg/kg. To generate this compound in *S. cerevisiae* with a dual purpose, to be a wine component or to industrially produce it (isolation and purification), a de novo pathway was generated [50]. The best combination and codon optimization for several genes (from different sources) was determined, and anaerobic/aerobic fermentations were carried out to prove the fitness of the strategy. The genes codified coumarate-CoA ligase (4CL), benzalacetone synthase (BAS), phenylalanine ammonia lyase (PAL), and cinnamate-4-hydroxylase (C4H), which allow the production of the ketone from the aromatic amino acid precursors phenylalanine and tyrosine. After testing the strain in grape juice, 0.68 and 3.49 mg/L of raspberry ketone were produced under anaerobic and aerobic conditions, respectively.

Additionally, volatile compounds such as monoterpenes (C_10_ class of terpenes) can play a part on it, but *S. cerevisiae* lacks an efficient route to produce these molecules. For instance, linalool provides a sweet floral alcoholic note. By functionally expressing the S-linalool synthase from *Clarkia breweri* in our yeast, geranyl diphosphate (GDP, present in the yeast) can be converted into linalool in microvinification conditions at least at twice its minimum perception threshold, without affecting growth or alcohol tolerance [46]. In another approach, heterologous expression of a geraniol synthase (GES) in the *S. cerevisiae* wine strain T_73_-4 in microvinification excreted ~0.75 mg/L geraniol de novo, an amount that surpasses 10-fold its olfactory threshold for perception [51]. Additionally citronellol, linalool, nerol, citronellyl acetate and geranyl acetate were detected as a further metabolization of geraniol of the yeast, with a final concentration of total monoterpenes concentration of ~1.56 mg/L, meaning a 230-fold increase. Monoterpenes have also been found to have antimicrobial, antiviral, anti-proliferative, antioxidative, anxiolytic, hypotensive or anti-inflammatory properties, having also an interest as nutraceuticals.

In some cases, acidic environments that are detrimental for yeast growth and lead to products with poorer taste, are required for the wine fermentation process. To try to solve this problem, *S. cerevisiae* yeast with higher tolerance to low pHs can be beneficial. To tackle this goal, atmospheric and room temperature plasma (ARTP) [52] to induce microbial mutation followed by high-throughput screening (HTS) and adaptive laboratory evolution (ALE) [53] were performed on *S. cerevisiae* ET008 for greengage plum wine production [54]. After 15 rounds of mutagenesis, five strains were selected which were in turn subjected to continuous cultivation with different pH values (3.0–2.5) for 90 days. The best survival rate at pH 2.5 was 95.5% which corresponded to ET008-c54, while for the parental it was only 9.73%. The main aromatic compounds were detected by GC-MS and the results indicated that the evolved strain ET008-c54 produced higher aromatic quality than the obtained in the parental; specifically, benzaldehyde is essential for the almond aroma in this type of wine, and its quantity in the new strain was twice that detected for the parental ET008.

A different approach to improve sensory qualities of wine is to carry out the fermentative process at low temperatures [55]. By culturing 27 commercial *S. cerevisiae* strains and introducing genetic variability by treatment with ethyl methanesulfonate, evolutionary engineering was performed [56]. After 200 generations at 12 °C, several strains reached twice the maximum OD (optical density) of the initial values and the full fermentative process was reduced from more than 720 h to 350 h. Metabolic changes detected as crucial for these variations were related to inositol and mannoprotein production.

**Figure 3:**
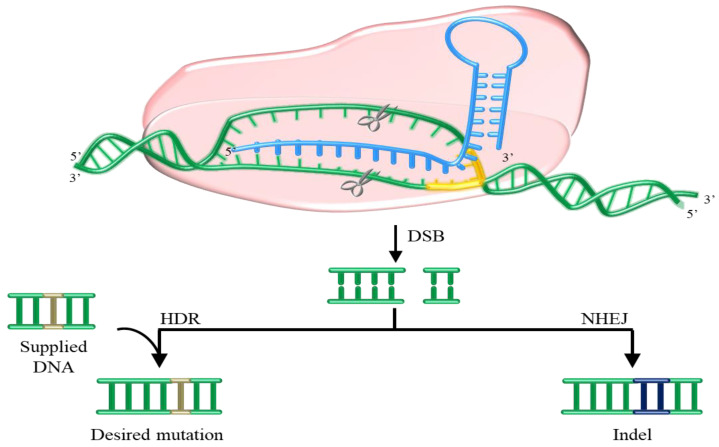
Scheme of the CRISPR/Cas9 editing mechanism. Cas9 protein (pink) and gRNA (light blue) get associated to the target DNA (green). By its nuclease activity, Cas9 produces a double-strand break (DSB) in the target sequence, guided by the PAM (protospacer adjacent motif, yellow). Then, the cell needs to repair the damage, by homologous direct repair (HDR) which can introduce controlled mutations by supplying a donor DNA, or by non-homologous end-joining (NHEJ), producing insertions or deletions (Indel).

The famous Clustered Regularly Interspaced Short Palindromic Repeats and associated protein nuclease system, CRISPR/Cas 9 in short, provides adaptive immunity against foreign nucleic acids to bacteria and archaea with the need of RNA-guided nuclease activity [57], and is broadly used as editing tool for researches, also in *S. cerevisiae* (Figure 3) [58,59,60]. The magnitude and relevance of this technique has been recognized with 2020’s Nobel Prize in Chemistry to Emmanuelle Charpentiere and Jennifer A. Douda (not less important is the work of Francisco Mojica, who discovered the basis of CRISPR). In a mixed properties approach, CRISPR/Cas9 was employed to overexpress the alcohol acetyltransferase 1 (ATF1, whose proteic product participates in acetate ester production) in *S. cerevisiae* AWRI 1631 [61]. The original promoter was substituted by a stronger one, specifically P_TEF1_. When fermentations were performed, the production of acetate ester (related to aroma) in the engineered strain spiked, mainly for isobutyl acetate with a ~15-fold increase. Since there is no foreign genetic material, the use of this strategy can ease the way to commercialize yeast for wine production with improved or ad hoc characteristics.

Dzialo and co-workers [62] have reviewed the principal pathways and genes related to aroma compounds formation that are present in yeast, which can be a sort of guide to target future approaches in wine engineering.

## 4. Ethanol

The concentration of ethanol influences several aspects of wine, not only regarding the process itself, in relation with the tolerance of the yeast (inhibiting growth when reaches ~110 g/L at 30 °C) [63], but also with the volatility of aroma compounds [64]. Of course it is also a matter of consumer preferences: some like it with the usual graduation, some like it “light” to keep enjoying a glass of wine in a healthier way. Additionally, there has been an increase in alcohol concentration of wine of ~2% (*v/v*) during the last two decades [65], due to climate change and a trend to late harvest in order to obtain wines with proper aromatic and phenolic maturity [66]. A higher alcohol graduation also implies higher taxes and regulation issues. In this section, approaches to vary the concentration of ethanol in the final product are reviewed.

### 4.1. Lower Ethanol Content

In order to produce wine with a reduced percentage of ethanol, redirection of the carbon flux during fermentation to give rise to different molecules than ethanol has been a recurrent approach. For instance, by introduction of a lactate dehydrogenase in yeast, pyruvate can be converted into ethanol and lactate [67]. However, these strategies create a metabolic imbalance that can affect the final quality of the wine [68]. To correct this detrimental side effect, further genetic engineering can be planned, such as the deletion of ALD6, encoding aldehyde dehydrogenase, to decrease acetic acid production [69] and overexpression of BDH1, encoding 2,3-butanediol dehydrogenase, to diminish acetoin generation [70]. Another work joined overexpression of glyceraldehyde-3-phosphate dehydrogenase gene (GPD1) and deletion of ALD6 to give rise to a *S. cerevisiae* strain, AWRI2531, which carried out fermentation with 1.5% *v/v* less ethanol than its parent [71]. Again, however negative effects in the quality of the beverage were generated. To avoid it, transcriptomics, proteomics proteomics and metabolomics were taken together so the main volatile metabolites and the genes implied responsible for the loss of good traits were identified. With this integrative approach gene remediation was achieved, and the results can be useful for future wine developments [72].

Another appropriate plan could be to divert the carbon flux to several metabolites. In a yeast engineering work, a NADH oxidase was expressed in *S. cerevisiae* so the NADH pool in the cell was reduced, which affected several redox reactions involved in various metabolic pathways [73]. The ethanol content was 15% lower than in the wild-type strain but its growth abilities were altered because of the accumulation of high levels of acetaldehyde. To solve this inconvenient, oxygen was supplied just during the stationary phase, with a 7% reduction in the ethanol content, but avoiding the negative effects in growth and fermentation [74].

A *S. cerevisiae* mutant strain named BY4743pdc2Δ519 was developed to reduce alcohol content in wine [75]. In this case, the performance of the pyruvate decarboxylase was modified by partial deletion of the gene PDC2, which codifies a transcription factor influencing its activity. The result was a reduction of 7.4% in the final ethanol content in lab scale-vinifications, while no negative effects were detected regarding residual sugar or volatile acidity. Moreover, when tested in a native and a commercial strain, the reduction achieved was 10% and 15%, respectively.

The reduction of the amount of glucose to be converted to ethanol in the fermentation process can be also taken as a strategy for reducing ethanol content. A way for this goal is to introduce in *S. cerevisiae* the gene encoding a glucose oxidase that leads to the production of H_2_O_2_ and gluconic acid [76]. This approach is limited for the low presence of oxygen demanded for the fermentation process and the fact that accumulation of gluconic acid is detrimental for the final quality of wine.

Other genes implied in trehalose biosynthesis, central glycolysis, the oxidative pentose phosphate pathway, and the tricarboxylic acid (TCA) cycle were noted to be potential targets for genetic engineering with the aim to obtain lower alcohol wines [77].

### 4.2. Higher Ethanol Content

For those wines requiring a higher content of ethanol, yeasts with increased tolerance towards this compound are beneficial. For instance, Chinese rice wine can reach 20% (*v/v*) ethanol, which negatively affects growth and metabolism, having as a consequence the sluggishness of the culture. In an adaptive laboratory evolution experiment aided by chemical mutagenesis with ethyl methane sulfonate, *S. cerevisiae* G85 was cultivated in sequential batch growth for 240 generations [78]. G85X-8 showed a death rate of 41.3% while for the parent was 93.5%, and the ethanol concentration reached in the wine fermentation process was 130 g/L vs. 126.6 g/L achieved for the wild-type. This strain was also found to display better tolerance to osmotic stress and temperature without significant differences in the final product. This effect was attributed to a higher presence of oleic acid and linoleic acid and lower proportions of myristic acid, a higher ratio in the plasma membranes of C_18_/C_16_ fatty acids, and a higher percentage of trehalose for the mutant G85X-8 compared with the wild-type.

In some cases, to achieve a determined phenotype in an organism, modification of one gene is not enough since multiple genes can be involved. Thus, by global transcription machinery engineering (gTME), transcription levels of several genes can be reprogrammed to obtain the desired traits [79]. In this sense, directed evolution of gTME has been used to increase ethanol tolerance in the *S. cerevisiae* wine strain Y01 [80]. SAGA (Spt-Ada-Gcn5 acetyltransferase) is a complement complex involved in transcription in *S. cerevisiae* which contains two TBP (TATA-box binding protein)-associated factors, SPT3 and SPT8 (suppressor of ty insertions) [81]. Mutations in SPT8 reduced cell vitality and increased cell death. A library was created by error prone PCR on SPT8 and screened with up to 16% ethanol. The best variant had an increased viability of 8.9%. Gene transcription analysis by real-time PCR revealed that SPT8 expression was 5.3-fold higher than in the original Y01.

## 5. Ethyl Carbamate

Ethyl carbamate (EC) is present in wines and since it was classified as probably carcinogenic for humans by the International Agency for Research on Cancer, reducing its content is rather convenient. Regulations worldwide have determined the maximum levels allowed in alcoholic beverages ranging from 30 µg/L in wine to 1000 µg/L in fruit spirits (European Food Safety Authority 2014:EN-578). Arginine is one of the most abundant amino acids in grape musts, and *S. cerevisiae* can transform it by an arginase, codified by CAR1, to ornithine and urea [82]. Then, urea can be metabolized by a yeast urea amidolyase encoded by the gene DUR1,2 [83] or it can chemically react with ethanol to form EC. Since arginine is the major source of EC (although not the only one), it is an interesting target for this goal. For instance, by overexpressing DUR1,2, a yeast with 89% less EC was developed [84]. In another recent engineering work, an arginase-deficient recombinant strain YZ22 was constructed by deleting CAR1 gene, which was able to reduce the formation of EC by 73.78% while its formation along storage was also slowed down, and no differences were detected in the growth and fermentation parameters with the parental strain [82].

Citrulline is a precursor of arginine, so its metabolism could also affect EC content. To determine if altering citrulline metabolism will reduce EC content, *S. cerevisiae* N85 strain was genetically engineered by disruption of ARG3 gene (its enzymatic product synthetize citrulline from carbamoyl phosphate and ornithine) or over-expression of ARG1/ARG4 genes (encoding two enzymes that transform citrulline in arginine) [85]. Unfortunately, the results in this case showed that the strategy followed is not appropriate to reduce the formation of EC during Chinese rice wine fermentation. We can take this case as an example of how intricate is the metabolism along the process and of the complexity of the task.

The CRISPR/Cas9 tool has also been exploited to reduce EC in wine by reducing urea in two yeast strains, EC1118 and AWRI796. CAN1 plasma membrane arginine permease is involved in the arginine degradation pathway that leads to urea production. This gene was eliminated by CRISPR/Cas9 and both strains showed a reduction in its capability to produce urea of 18.5% and 35.5%, respectively [86]. When wine production conditions were mimicked, again both engineered yeasts failed to produce urea while fermentation was completed after 8–12 days. The advantages of CRISPR/Cas9 were also used to further engineer a previously genetically manipulated yeast strain in which overexpression of DUR1,2 was carried out. DUR3 was also overexpressed giving rise to a yeast strain with a higher capacity to use urea, reducing its amount in 92% and avoiding in this way the formation of 58.5% EC [87].

The ALE strategy has also been used in order to diminish the amounts of urea in wine. *S. cerevisiae* XZ-11 was cultivated along 360 rounds of transfer with urea as the sole nitrogen source to apply selective pressure towards its utilization [88]. A selected strain was able to decrease the residual urea concentration to a 43.9% when using 0.6 g/L in the initial medium, and 47.9% when wine fermentation conditions were applied. For EC, the levels were 40.5% lower with this variant than those for the parental type, all of this without varying the volume percent of ethanol in the final product. The variant also showed high genetic stability after six generations. Sequence analysis revealed fast evolving genes such as AAT2, (transformation of α-ketoglutarate to glutamate), PRO1 and ARG2 (both involved in the conversion of glutamate to ornithine).

Can1p, Gap1p and Alp1p are amino acid permeases involved in arginine intake into the cells. In another engineering work, seven combinations of arginine permeases gene disruption were tested. When the mutant strains were grown in yeast nitrogen base plus the 20 amino acids, those with the disrupted CAN1 gene reduced by 80% the concentration of extracellular urea [89]. When the broth used was YPD, more similar to wine fermentation, the variant with disrupted CAN1 and GAP1 genes had a 68% lower extracellular urea. Moreover, since Gln3p is a nitrogen catabolite repressor (NCR) regulator acting in the activation of NCR-sensitive genes transcription [90], a truncated Gln3p was overexpressed in the different variants, and those with disrupted GAP1 + CAN1, and ALP1 + GAP1 + CAN1 reduced to a higher extent the presence of extracellular urea (67% and 32%, respectively).

## 6. Other Oenological Traits

### 6.1. Oxidative Stress

One of the molecules that helps to reduce the oxidative stress is glutathione (GSH), together with other molecules and enzymes (such as trehalose, catalase, superoxide dismutase, and glutathione reductase) [91], which lengthens the preservation of the precious wine properties. By performing random genetic recombination of 69 clones of the *S. cerevisiae* strain UMCC 855, a GSH high-producer variant was disclosed, which in turn showed a strong fermentative aptitude [92]. In a similar approach, two evolved strains were found that increased GSH presence in wine of by up to 100% compared with the parental strain [93].

Proteins like superoxide dismutases Sod1p and Sod2p, and Hsp12p play a part in preventing oxidative stress, so overexpression of the three genes SOD1, SOD2, and HSP12 was carried out in three *S. cerevisiae* strains, leading to variants with improved specific activity and higher levels of GSH peroxidase and reductase activities, together with a higher intracellular GSH content and lower peroxidized lipid concentration [94]. These strains developed the velum faster and it was of a higher quality, which can speed up metabolism and wine aging, lowering the period for wine maturation, an interesting improvement for reducing expenses.

### 6.2. Mannoproteins

Proteins with a high content in mannose-type sugars are known as mannoproteins (30% peptides, 70% sugar residues, of which 98% are mannose) and are found in yeast cell walls. When wines are aged in lees, components of the cells are incorporated to the product, for instance by autolysis, providing different qualities. Mannoproteins are of important influence in this fact, and have been proved to be implied in increasing the wine heat and cold stabilities, aid the growth of malolactic bacteria, influence aromatic contents of wine, have effects on organoleptic characteristics such as gustatory sensations of texture, roundness and mouthfeel, and are beneficial for the formation and stabilization of sparkling wine foams [95]. Since Knr4p is involved in coordinating the synthesis of the cell wall and the emergence of the bud, the effects of deletion of KNR4 in *S. cerevisiae* have been tested with regard to mannoprotein occurrence in wine making. An increase in the freed mannoproteins was observed in the strain EKD-13 (obtained from EC1118) [96,97]. Further analysis suggested that neither autolysis nor overexpression of mannoprotein codifying genes were responsible of this phenotype, but the inability of Knr4p to perform its coordination function leading to a failure in the anchoring of the mannoproteins to the cell wall [98].

### 6.3. Osmotic Stress

Some juices, such as icewine juice, can have up to 340 g/L of sugars [99], producing hyperosmotic stress in *S. cerevisiae* during wine fermentation. To fight it, yeasts can produce glycerol or take it from the extracellular environment by H^+^/glycerol symporter Stl1p [100]. In order to determine Stl1p’s effect in such situations, CRISPR/Cas9 was used in the commercial yeast K1-V1116 to eliminate the full open reading frame of its encoding gene [101]. When this modified yeast was cultured in icewine juice, the growth was slower and less biomass was achieved than in the case of its parental; additionally, elevated glycerol and acetic acid production was detected, showing how Stl1p is involved in osmotic stress and signaling this symporter as a target for improvement of strains in this conditions.

### 6.4. Sulfur Compounds

Sulfur dioxide, SO_2_, is usually added to the wine fermentation process because of its antioxidant properties and in order to avoid the growth of unwanted microorganisms from the starter culture that can spoil the process [102]. This molecule dissociates into bisulfite (HSO_3_^−^) and sulfite (SO_3_^2−^) once it enters a cell [103], and their negative effects in the yeast include depletion of ATP, damage of the plasma membrane, proteins, vitamins and coenzymes [104,105,106]. Legal levels of SO_2_ are limited to up to 400 mg/L, a concentration that some spoilage microorganisms can tolerate [107]. The sulfite efflux pump Ssu1 is known to have a key role in *S. cerevisiae* defense towards sulfite [108,109], and recently, the transcription factor Com2 was analyzed for this function. The *S. cerevisiae* strain BY4741 was used as a parental type to create a COM2 deleted mutant [107]. Transcriptomics and genome-wide phenotypic analyses were conducted that showed that Com2 controls the expression of more than 80% of the genes activated by SO_2_ and the cell viability is compromised. These results suggest that protection against SO_2_ includes the sulfate reduction pathway genes and most of the genes employed in lysine biosynthesis and arginine. This work also demonstrated that lysine supplementation can diminish toxic effects of SO_2_ in wine yeast.

The CRISPR/Cas9 technique has also been applied to efficiently engineer uniquely targeted and multiple reciprocal translocations, in order to characterize the implication of structural variants in phenotypic diversity regarding different conditions and genetic background [110]. This work disclosed the fact that when a translocation found in wine isolates (SSU1/ECM34, from a recombination event between four base-pair micro-homology regions on chromosomes VIII and XVI) was reproduced in the laboratory strain BY4741, sulfite resistance was lower in the laboratory strain than for the wine isolate. Promoter repeats at the SSU1 gene were added to the engineered strain, showing an increased resistance, but still lower than that showed by wine isolates. This is an example of how tricky it can be to transfer conclusions between laboratory and wine yeast strains and conditions.

Regarding H_2_S, its apparition during wine fermentation is due to *S. cerevisiae* metabolism, with the consequent unwanted off-flavors. In a highly successful work, mutations were introduced in MET10 and MET5 genes (encoding α- and ß-subunits of the sulfite reductase enzyme) in the commercial wine yeast PDM, resulting in a H_2_S generation reduced up to 99% in synthetic and also in grape juice fermentations, without detecting other differences in chemical composition [111].

### 6.5. Low Temperature Fermentation

As it has seen above, carrying out the fermentation of the must at low temperature is a proper strategy to improve organoleptic properties of the final beverage, but this condition can also help the yeast when facing low pH, high osmotic pressure, or low nitrogen accessible in grape must [112]. The psychrotolerant *S. cerevisiae* AXAZ-1 strain has been used to investigate and develop intense aromatic wines [113,114], while it represents an interesting prospect for genomic analysis to elucidate the mechanisms responsible of such an advantageous capacity. In an effort to elucidate genetic determinants involved in good performance at low temperature, quantitative trait loci (QTLs) were mapped in the *S. cerevisiae* commercial strains P5 (Lalvin^®^ICVGRE) and P24 [115]. By crossing the two of them, populations of segregants were obtained in 13 generations, the results pointing at regions in chromosomes XIII, XV and XVI (subtelomeric regions) and in chromosome XIV as responsible for low temperature adaptation, opening new possibilities for genetic engineering. In another approach, QTL on chromosome I was spotted as related to the maximal fermentation rate at low temperature, and the FLO1 gene was found to be involved in this effect [116]. Additionally, genes involved in translation efficiency have been disclosed as responsible of limiting cold adaptation [112], while the study of knockouts in *S. cerevisiae* BY4743 pointed at AHP1, MUP1, and URM1 genes as implied in low temperature growth capacities [117].

### 6.6. Nitrogen Usage

Another essential target for the betterment of the final product and the efficiency of the process is the nitrogen consumption by *S. cerevisiae*. Although this element is present in the grape juice, it commonly acts as a limiting factor since not all nitrogen sources are well assimilated by the yeast, which causes low biomass and sluggish or stuck fermentative process [118]. In this regard, a term to have in mind is YAN (Yeast Assimilable Nitrogen), which is usually formed by amino acids (mainly glutamine, glutamate and asparagine) and ammonium [119]. The methods employed to avoid this handicap are the fertilization of the grapevine or the addition of nitrogen sources to the must, both of them having negative consequences in the costs and the production of off-flavors and toxic compounds such as EC [119]. Biotechnology and genetic engineering, once again, come to the rescue in this matter. In selection experiments in which SGRP-4X population was employed (four strains of yeast lineages—North American, Sake, West African and Wine European—were outcrossed for 12 generations giving rise to the SGRP-4X population, ~10–100 million random segregants) the ECM38 allele from the North American strain was detected as the effector of the best growth rate when nitrogen-limited conditions were used [120]. The TORC1 pathway has been seen to act as the main mechanism in nitrogen-limiting adaptation (amongst other genetic elements), which situates the genes involved as interesting targets for modifications to this end [118,121,122,123,124,125].

## 7. GMOs and Non-GMOs

When taking in consideration the real commercial implementation of all these and other advances, a barrier arises: engineered yeasts are usually considered genetically modified organisms (GMO) and legal issues impede their use. To the best of our knowledge, so far only two strains have been allowed for commercial implementation [84,126], although they are not extendedly used. In the European Union, Regulation (EC) 1829/2003 sets the legislation on genetically modified food and feed.

The laboratory created yeast strains that are not subject to these specific and complicated regulations are those obtained by random mutagenesis, interspecific and intraspecific genetic hybridization, rare mating, or experimental evolution (ALE) [5]. In the case of the so-called self-cloned organisms (without heterologous DNA, e.g., promoter usage), yeast strains were allowed for use in beverage production in Japan [127], so this strategy can help the commercialization of modified strains [128].

An interesting alternative to explore genetic variability without creating GMOs is to take advantage of genes with an outstanding easiness to mutate spontaneously, such as the flocculins encoding FLO family. This behavior is due to their subtelomerical position and tandem repeats [129]. Flocculins are of interest for winemakers because they help in the clarification of wine, and act protectively when the conditions of the environment are harsh [130].

Additionally, public opinion on GMOs is crucial to the success of engineered yeasts, and it is far from being positive. Public opinion and legislation are intertwined and influence each other, so for the wine industry (and food and beverage industries in general) to benefit from all the efforts in the field, both should move forward, without compromising human nor Earth’s safety.

## 8. Conclusions

There is an actual preference for quality rather than for amount, meaning that in the demanding for premium wines, less is more. Here, different wine traits that can be modulated by genetic engineering have been reviewed and exemplified (Figure 2), but this technology can be exploited for winemaking in a “virtually infinite process” to adapt the product to the changing times [92]. To this challenge, some other relevant biotechnological techniques (and, of course, non-biotechnological) are used, such as hybridization, but are not described here since they imply non-*S. cerevisiae* species, and have been reviewed elsewhere [5,131]. In todays globalized and 2.0 world, where news are spread at the speed of light, public opinion can determine waves of “dos and don’ts” and legislation is constantly being updated, it is preferable to conduct research for non-GMOs. Additionally, a vast effort is required to develop completely safe GMOs, not only for the sake of economic interests, but for the sake of progress. These organisms can help us in countless aspects of life, not all of them as hedonist as enjoying a glass of wine, but related to health, economy, poverty or environment.
